# A Potential Link Between Outcome of Periodic Fever, Aphthous Stomatitis, Pharyngitis, Cervical Adenitis (PFAPA) Syndrome in Children and Breastfeeding: A Retrospective Single-Center Cohort Snapshot

**DOI:** 10.3390/children11121559

**Published:** 2024-12-23

**Authors:** Donato Rigante, Marcello Candelli

**Affiliations:** 1 Department of Life Sciences and Public Health, Fondazione Policlinico Universitario A. Gemelli IRCCS, 00168 Rome, Italy; 2 Periodic Fever Research Center, Università Cattolica Sacro Cuore, 00168 Rome, Italy; 3 Department of Emergency Anesthesiologic and Reanimation Sciences, Fondazione Policlinico Universitario A. Gemelli IRCCS, 00168 Rome, Italy; marcello.candelli@policlinicogemelli.it

**Keywords:** PFAPA syndrome, child, autoinflammation, personalized medicine, recurrent fever, breast milk, innovative biotechnologies

## Abstract

*Background/Objectives:* Periodic fever, aphthous stomatitis, pharyngitis, and cervical adenitis syndrome, often referred to as PFAPA syndrome, may enigmatically recur for an undetermined time in affected children: a potential reason to explain its recurring pattern for an unpredictable period or its self-limitation is currently unknown. We explored the relationship between different general, demographic, clinical, and laboratory features of PFAPA children and disease evolution over the course of a decade. *Methods:* We have retrospectively screened 150 Italian children with a history of PFAPA syndrome attending the Outpatients Clinic of Pediatric Rheumatology in our Institution during the period 2014–2024, all without any recognized chronic diseases: 88 males, 62 females, mean age at onset of 2.5 ± 1.7 years, age range of 0.3–9.4 years, and mean age at diagnosis of 4.5 ± 2.0 years. The whole cohort of PFAPA patients had been followed up for a median period of 5 years (IQR: 4–7). *Results and Conclusions:* After dividing patients into two groups based on either the disappearance or persistence of PFAPA symptoms during follow-up, we found that positive family history of recurring fevers, cervical lymphadenopathy, arthralgia, myalgia, and breastfeeding for more than 6 months were associated with the disappearance of febrile attacks for at least six months. Performing a multivariate analysis adjusted for sex and age, we found that only breastfeeding duration longer than 6 months and higher education level of PFAPA patients’ mothers were independently associated with the resolution of PFAPA symptoms.

## 1. Introduction

The “periodic fever, aphthous stomatitis, pharyngitis and cervical adenitis” (PFAPA) syndrome is a shadowy disorder of childhood, which can be defined by the combination of periodically recurring fevers with nonspecific mucosal signs as oral aphthosis and pharyngitis (either exudative or non-exudative) and cervical lymph node enlargement [1]. There is no specific diagnostic test to strictly identify PFAPA children, and the disease can be suspected through the handful of “classical” clinical signs drafted by Marshall in 1987 from the Vanderbilt University Medical Center, then modified by Thomas in 1999, requiring that febrile attacks start within 5 years of age and that patients should not display any respiratory tract infection along with fevers, should be completely symptomless between febrile episodes, and should have unimpaired growth [2]. A careful reconstruction of family history, considering the patient’s ethnicity, nontypical symptoms suggesting specific causes of fever, and results of laboratory tests that turn to normal between PFAPA episodes can help sustain PFAPA diagnosis and exclude the many other causes of recurrent fevers in a child [3]. The evolution of the syndrome remains unpredictable in the long run.

The pathogenesis of PFAPA syndrome is not yet deciphered, warranting the hypothesis of a multifactorial origin probably based on a polygenic pattern of susceptibility; its treatment relies on the administration of one-shot low-dose corticosteroid at the febrile attack, which promptly aborts the flare, but cannot prevent the subsequent recurring fever episodes over an unpredictable lapse [4,5]. Tonsillectomy has also proved to be successful in some pediatric patients, but a larger experience confirming its efficacy is needed to tilt toward a primary surgical approach in PFAPA children [6]. Furthermore, the increasing number of reports of PFAPA syndrome in adults has emphasized the misconception that this disorder could be limited to sole pediatric patients, and various scores have been developed to try to exclude hereditary autoinflammatory disorders [7,8].

In this study related to a single-center cohort of children with a confirmed diagnosis of PFAPA syndrome, who were longitudinally assessed in the long term, we have retrospectively evaluated their medical charts to define which general, demographic, clinical or laboratory clues might potentially influence the time required for disease resolution, i.e., a full PFAPA disappearance, or predict disease persistence with a recurrent pattern over time.

## 2. Patients and Methods

We have retrospectively evaluated the medical charts of 153 Italian children with history of recurrent fevers in the Outpatients Clinic of Pediatric Rheumatology of our Institution, all without any evidence of chronic diseases, immune deficiencies, or autoimmune disorders, who satisfied Marshall/Thomas and the EuroFever criteria for diagnosing PFAPA syndrome during the period 2014–2024. The exclusion of hereditary monogenic autoinflammatory disorders by genetic analysis was performed in 25 patients (16.3% of the initial cohort) based on their ethnicity, past familiar history of recurrent fevers in parents and relatives, or previous immunological investigations. During the follow-up evaluation, two patients were excluded from the cohort, as they were found to have the P369S/R408Q *MEFV* mutations (consenting to accordingly diagnose familial Mediterranean fever) and the V377I/G347V *MVK* mutations (consenting to accordingly diagnose mevalonate kinase deficiency), respectively. One further patient was excluded from the cohort as she developed signs of autoimmune lymphoproliferative syndrome. No pathogenic mutations were found in the remainder of the genetically tested PFAPA patients.

### 2.1. Inclusion of PFAPA Patients for the Retrospective Assessment

A total of 150 patients from the initial cohort were included in our assessment: 88 males and 62 females, mean age at onset of 2.5 ± 1.7 years, age range of 0.3–9.4 years, and mean age at diagnosis of 4.5 ± 2.0 years. The study was conducted according to guidelines of the Declaration of Helsinki and was approved by the Local Ethical Committee as part of a larger protocol based on the evaluation of patients with complex or rare diseases (approval code from our Ethical Committee: 2105; approval date: 5 February 2019): all patients’ parents and/or their legal guardians signed an informed consent for this retrospective assessment. General, demographic, clinical, and laboratory data were collected by analyzing patients’ medical charts: for each patient, we longitudinally recorded anthropometric data at diagnosis (to exclude cases with growth delay referred to systemic conditions), duration of fever episodes, duration of intervals between flares, and clinical manifestations occurring during attacks. Serial thorough investigations were periodically performed in all cases displaying a recurrent disease. Infant birth weight, auxological characteristics of patients, and vitamin supplementation during the period of PFAPA diagnosis were not considered by the study.

### 2.2. Data Related to Maternity Care and Breastfeeding

The medical charts of PFAPA patients were used to retrieve further information related to mothers of each patient: maternal demographic and socio-economic characteristics, including ability to breastfeed their daughters or sons, and direct data about breastfeeding. No child in the cohort had any previous congenital malformations that might interfere with breastfeeding. Exclusive breastfeeding implied that the infant received only breast milk (without intake of non-human milk or other liquids), though allowing the child to receive vitamin supplementation. We also assessed the education level of mothers, dividing them into two groups: high school diploma or graduation as having a high level of education, and middle school diploma or lower as having a low level of education; we chose this simple division to avoid comparing groups with smaller numbers of individuals. Mothers’ age, gestational age of pregnancy, modes of delivery, and singleton or twin pregnancy were not evaluated by this assessment. Also, mothers’ employment, marital status, type of maternity hospital (in residential or in country areas), body mass index before pregnancy, and previous breastfeeding experience were not considered, as well as smoking habits and caffeine or substance abuse before conception, antepartum, and postpartum. Any eventual correlations with the COVID-19 pandemic (during the period 2020–2022) were not considered.

### 2.3. Statistical Analysis Performed in the Study

The statistical analysis was performed using the SPSS version 25. Continuous data were presented using the mean and standard deviation for normally distributed data and the median and interquartile range for non-normally distributed data. Dichotomous data were described as counts and percentages of the total. To compare continuous variables, we used the Student’s *t*-test (for normally distributed data) and the Mann-Whitney test (for non-normally distributed data). To compare prevalence, we used the chi-square test or Fisher’s exact test (the latter if the count in a group was less than 6). We also performed a multivariate analysis using multinomial logistic regression, including all variables that had a *p*-value less than 0.15 at the univariate analysis and adjusting data for sex and age. A *p* less than 0.05 was considered statistically significant.

## 3. Results

The whole cohort of PFAPA patients had been followed-up at the Outpatients Clinic of Pediatric Rheumatology of our Institution for a median period of 5 years (IQR: 4–7): in particular, patients who displayed the resolution of PFAPA syndrome, i.e., the disappearance of febrile attacks for at least six months, had been followed-up for a median period of 6 years (IQR: 5–9), while those having persistent PFAPA symptoms were followed-up for a median of 5 years (IQR: 4–6.75).

Further information about the management of these children was retrieved by medical records: 143 of the cohort (95%) had been treated with one-shot low-dose corticosteroids (given during the febrile attack), and this strategy led to disease disappearance (no further flare-up for a minimum follow-up of 6 months) in 125 (83%), with no mid/long-term sequelae. Tonsillectomy was performed in nine patients (6% of the entire cohort) who had reported no long-term benefit from on-demand steroids, leading to PFAPA resolution in seven of them (78%). Finally, since 2019, 23 patients (15.3% of the whole cohort) were also treated with oral supplementation of *Streptococcus salivarius* K12, irrespective of on-demand steroid administration, as the prophylaxis with this probiotic given for at least 6 months was found to mitigate PFAPA febrile attacks [9]. *Streptococcus salivarius* K12 favored a complete remission in 15 out of 23 patients (65%).

After dividing PFAPA patients into two groups based on the disappearance or persistence of PFAPA symptoms for at least six months during the follow-up period, we found that family history of recurring fevers, presence of cervical lymphadenopathy, presence of arthralgia and myalgia during attacks were associated with persistence of the syndrome. Conversely, patients who were breastfed for more than 6 months showed a significantly higher percentage of FPAPA symptom resolution compared to those who were breastfed for a shorter period [115/132 (87%) versus 3/18 (17%), respectively; *p* < 0.0001; see Table 1].

Performing a multivariate analysis adjusted for sex and age, we found that only breastfeeding for more than 6 months and a higher maternal education level remained independently associated with the disappearance of PFAPA symptoms (see Table 2).

## 4. Discussion

Breast milk serves as a powerful link between maternal and offspring health, fueling a biological interdependent relationship that impacts a child’s immunological development and giving rise to reinforced protection against diseases [10]. Key findings from the literature highlight that longer duration of breastfeeding is associated with numerous health benefits, including a reduced risk of gastrointestinal or respiratory infections, better growth and cognitive development, and a lower likelihood of developing allergic diseases and obesity in life [11]. While numerous studies suggest various positive effects of a longer breastfeeding, the quality and reliability of these findings are often compromised by methodological limitations and insufficient control for confounding variables.

Many gaps exist in the medical literature related to the interaction of environmental factors with different systemic autoinflammatory conditions, which represent a rising spectrum of diseases stamped by dysregulation of the innate immune system, including PFAPA syndrome, being the most frequent multi-factorial nongenetically-based autoinflammatory disorder [12]. There is no infectious agent etiologically connected with PFAPA syndrome, though many PFAPA children are treated with antimicrobials during febrile attacks. Moreover, in PFAPA attacks, there is increased expression of various inflammatory cytokines, especially interleukin (IL)-1β, as a result of inflammasome overactivity [13]. The outcome of PFAPA syndrome is unpredictable, but the disease generally resolves by itself over a variable period of time with no sequelae, though lasting in a few cases until adulthood: the recurring PFAPA clinical picture may depend on rhythmic variations in physiological processes mediated by both endogenous and exogenous factors as changes in the ratio of microflora elements of the oropharyngeal mucosa [14]. 

It is indisputable that early intestinal flora in children is influenced by gestational age, mode of delivery, or nutrition habits, all evoking a regular microbiome functional development, and that gut microbiota elicits effects on both promotion of entero-endocrine signaling and innate immunity within the gut epithelial barrier [15]. Environmental factors may be important to both the pathogenesis and evolution of PFAPA symptoms: a Finnish case-control study showed that mothers of PFAPA children had lower breastfeeding rates in comparison with controls (94 versus 99%, *p* = 0.006) [16]. Moreover, breastfeeding has been linked to the development of a specific microbiota ‘within’ the mouth, ensuring local immunity with proactive commensal flora and beneficial host-flora interactions with protection against immune-mediated diseases [17]. Na et al. performed a nationwide cohort study in South Korea to explore the relationship between various factors and Kawasaki disease, including almost 2,000,000 infants: the most prevalent feeding type was exclusive breastfeeding (41.5%) and, at the 1-year follow-up, only 3854 (0.2%) of these infants had developed Kawasaki disease [18]. Guo et al. performed a retrospective cohort study related to 249 children with Kawasaki disease, analyzing breastfeeding rates, and found that longer breastfeeding was associated with a shorter duration of fever and lower risk of Kawasaki disease-related coronary artery abnormalities [19]. A Turkish study evaluated 182 patients diagnosed with acute rheumatic fever between 2010 and 2019, divided into groups according to the presence of carditis; patients’ demographic, socio-economic, and breastfeeding data were compared between the groups, and breast milk intake for less than 6 months was shown to be an independent predictor of carditis occurrence [20]. Therefore, breastfeeding may be considered a cornerstone for child’s healthy development, providing crucial nutritional components and numerous biologically active molecules for microbiota settlement [21]. 

Nevertheless, barriers to adhering to breastfeeding recommendations are numerous, resulting in a lack of initiation or premature cessation of breastfeeding. Not coincidentally, the World Health Organization European Regions report the lowest rates of exclusive breastfeeding in relationship to socio-cultural issues, characteristics of the healthcare services, institutional policies, and public health frameworks [22]. Many multifaceted biological compounds, such as bioactive molecules and exosomes contained within the human breast milk, boost immune and metabolic system development, supporting the long-term health of children through protective mechanisms and even epigenetic changes, which may significantly reinvigorate innate immunity [10,11]. So far, the association between the duration of exclusive breastfeeding and onset or evolution of PFAPA syndrome has not been elucidated. After all, the clockwork periods of PFAPA febrile attacks might derive from a rhythmic défaillance in the regulation of innate immunity within the oral cavity, which periodically recur due to the disrupted recognition of resident microbial communities as commensals, triggering an autoinflammatory response [23].

In general terms, the PFAPA syndrome recovers on its own, but children who do not manifest a tendency to remission after “on demand” one-shot corticosteroid therapy may be addressed to tonsillectomy, while the use of *Streptococcus salivarius* K12 given per os may represent a further suitable therapeutic tool. Our retrospective study suggests that breastfeeding for more than 6 months and higher level of mother’s education can be associated with self-resolution of PFAPA syndrome for children who will develop this disorder during infancy. It is well-known that breastfeeding has been associated with a mother’s educational level, and women with higher education levels are more likely to practice exclusive breastfeeding for 6 months [24]. In conclusion, our study is a simple snapshot related to children affected with PFAPA syndrome, which has several limitations:

(a) Retrospective nature of the study and lack of adequate control for different confounding factors;
(b) Various factors impacting breastfeeding initiation and duration were not considered, including the child’s birth weight, mother’s body mass index, promotion of different feeding practices following midwife-led antenatal breastfeeding counseling, or any eventual support during the breastfeeding period;
(c) No information related to smoking or substance abuse by mothers;
(d) The interpretation of the study results is limited by the relatively small sample size;
(e) Auxological characteristics of patients were not considered by the study;
(f) Any eventual correlations with the COVID-19 pandemic during the period 2020–2022 were not considered.

Furthermore, much of the scientific data available comes from studies conducted several years ago; these older studies may not reflect current breastfeeding practices or advancements in research methodologies. Of course, there is a need for updated and comprehensive studies to validate and extend the findings of our study. A future goal of research might be a better evaluation of differences in breastfeeding indicators, which may ostensibly influence the outcome of PFAPA syndrome during childhood.

## 5. Conclusions

The overall gut microbiota composition has been correlated with different childhood diseases, but the exact microbial contributions to the origin of many of such disorders remain unclear. Environmental factors can be important in the evolution of PFAPA syndrome, but they should be further evaluated by future studies considering larger samples of patients from different countries and cultures, addressing also confounding factors, to help clinicians and mothers optimize the infant’s microbiota throughout infancy. Our study reveals that breastfeeding for more than 6 months can be associated with self-resolution of PFAPA syndrome, while the multivariate analysis adjusted for sex and age shows that only exclusive breastfeeding for more than 6 months and higher education level for PFAPA patients’ mothers are independently associated with the disappearance of symptoms for at least six months.

## Figures and Tables

**Table 1:** Basic general/demographic data, clinical features occurring during febrile attacks, and treatments given to the cohort of patients with PFAPA syndrome retrospectively examined in the study.

	All Patients (*N* = 150)	PFAPA Syndrome Resolved (*N* = 132)	PFAPA Syndrome Not-Resolved (*N* = 18)	p *
Age at onset (years, m ± SD)	2.5 ± 1.7	2.5 ± 1.7	3.0 ± 1.5	0.32
Males [N (%)]	88 (59)	78 (59)	10 (56)	0.77
Females [N (%)]	62 (41)	54 (41)	8 (44)	0.77
Age at diagnosis (years, m ± SD)	4.5 ± 2.0	4.4 ± 2.0	4.9 ± 2.0	0.51
Familiarity for history of recurring fevers [N (%)]	45 (30)	36 (27)	9 (50)	0.05
Higher level of education in mothers [N (%)]	86 (57)	72 (55)	14 (78)	0.08
Breastfeeding for more than 6 months [N (%)]	118 (79)	115 (87)	3 (17)	0.0001
Fever duration in each attack in days (m ± SD)	4.0 ± 1.1	3.9 ± 1.1	4.3 ± 1.1	0.11
Days of fever in one year (m ± SD)	52.5 ± 18.4	52.4 ± 18.1	53.2 ± 20.8	0.89
Free-disease interval in weeks [M (IQR)]	4 (3–4)	4 (3–4)	4 (4–6)	0.24
Febrile attacks per year [M (IQR)]	12 (12–15)	12 (12–15)	12 (9.5–13.5)	0.22
Oral aphthosis [N (%)]	82 (55)	74 (56)	8 (44)	0.35
Pharyngitis [N (%)]	147 (98)	130 (98)	17 (94)	0.32
Exudative pharyngitis [N (%)]	91 (61)	79 (60)	12 (67)	0.58
Non-exudative pharyngitis [N (%)]	56 (37)	51 (39)	5 (28)	0.44
Cervical lymphadenopathy [N (%)]	113 (75)	96 (73)	17 (94)	0.05
Asthenia [N (%)]	21 (14)	18 (14)	3 (17)	0.72
Abdominal pain [N (%)]	62 (41)	54 (41)	8 (44)	0.77
Gastrointestinal symptoms [N (%)]	21 (14)	19 (14)	2 (11)	1
Headache [N (%)]	36 (24)	30 (23)	6 (32)	0.32
Arthralgia [N (%)]	65 (43)	51 (39)	14 (78)	0.002
Myalgia [N (%)]	56 (37)	43 (32)	13 (72)	0.002
One-shot corticosteroid administration [N (%)]	143 (95)	124 (94)	18 (100)	0.94
Tonsillectomy [N (%)]	9 (6)	7 (5)	2 (11)	0.29
Administration of *Streptococcus salivarius* K12 [N (%)]	23 (15.3)	15 (11.4)	8 (55.6)	0.0001

N: number; m: mean; SD: standard deviation; M: median; IQR: interquartile range. * *p* values have been calculated using Student *t* test for variables expressed in mean and SD, Mann Whitney U test for variables expressed in median and IQR, and chi-square test for variables expressed in numbers and percentages.

**Table 2:** Multiple logistic regression analysis adjusted for sex and age dealing with the variables associated with resolution of PFAPA syndrome in children retrospectively examined in the study (all variables with *p* ˂ 0.15 at the univariate analysis were included).

Variable	p *	OR (95%CI)
Sex	0.75	1.267 (0.289–5.557)
Age	0.59	1.109 (0.765–1.606)
Fever duration in each attack (days)	0.85	0.932 (0.444–1.958)
Higher level of education in mothers	0.05	5.432 (1.023–26.829)
Familiarity for history of recurring fevers	0.48	0.547 (0.107–2.785)
Breastfeeding for more than 6 months	0.0001	35.273 (6.026–206.472)
Cervical lymphadenopathy	0.06	0.073 (0.005–1.089)
Arthralgia	0.26	0.263 (0.037–3.523)
Myalgia	0.35	0.354 (0.040–3.113)
*Streptococcus salivarius* K12 administration	0.24	0.387 (0.078–1.910)

CI: confidence interval; OR: odds ratio; high mothers’ education level: achievement of higher school diploma or graduation. * p values have been calculated using a multiple logistic regression analysis.

## Data Availability

Data are contained within the article.

## References

[1] Rigante D. (2020). When, how, and why do fevers hold children hostage?. J. Evid.-Based Med..

[2] Manthiram K. (2023). What is PFAPA syndrome? Genetic clues about the pathogenesis. Curr. Opin. Rheumatol..

[3] Rigante D., Frediani B., Galeazzi M., Cantarini L. (2013). From the Mediterranean to the sea of Japan: The transcontinental odyssey of autoinflammatory diseases. BioMed Res. Int..

[4] Okamoto C.T., Chaves H.L., Schmitz M.J. (2022). Periodic fever, aphthous stomatitis, pharyngitis and cervical adenitis syndrome in children: A brief literature review. Rev. Paul. Pediatr..

[5] Takeuchi Y., Shigemura T., Kobayashi N., Nagumo H., Furumoto M., Ogasawara K., Fujii H., Takizawa M., Soga T., Matoba H. (2019). Clinical features and new diagnostic criteria for the syndrome of periodic fever, aphthous stomatitis, pharyngitis, and cervical adenitis. Int. J. Rheum. Dis..

[6] Ohnishi T., Sato S., Uejima Y., Kawano Y., Suganuma E. (2022). Periodic fever, aphthous stomatitis, pharyngitis, and cervical adenitis syndrome: Clinical characteristics and treatment outcomes—A single center study in Japan. Pediatr. Int..

[7] Rigante D., Vitale A., Natale M.F., Lopalco G., Andreozzi L., Frediani B., D’Errico F., Iannone F., Cantarini L. (2016). A comprehensive comparison between pediatric and adult patients with periodic fever, aphthous stomatitis, pharyngitis, and cervical adenopathy (PFAPA) syndrome. Clin. Rheumatol..

[8] Cantarini L., Iacoponi F., Lucherini O.M., Obici L., Brizi M.G., Cimaz R., Rigante D., Benucci M., Sebastiani G.D., Brucato A. (2011). Validation of a diagnostic score for the diagnosis of autoinflammatory diseases in adults. Int. J. Immunopathol. Pharmacol..

[9] La Torre F., Sota J., Insalaco A., Conti G., Del Giudice E., Lubrano R., Breda L., Maggio M.C., Civino A., Mastrorilli V. (2023). Preliminary data revealing efficacy of *Streptococcus salivarius* K12 (SSK12) in Periodic Fever, Aphthous stomatitis, Pharyngitis, and cervical Adenitis (PFAPA) syndrome: A multicenter study from the AIDA Network PFAPA syndrome registry. Front. Med..

[10] Lynch H.F., Greiner D.J., Cohen I.G. (2020). Understanding the mother-breastmilk-infant “triad”. Science.

[11] Froń A., Orczyk-Pawiłowicz M. (2024). Breastfeeding beyond six months: Evidence of child health benefits. Nutrients.

[12] Gardner N.J. (2023). PFAPA syndrome in children. J. Am. Acad. Physician Assist..

[13] Stojanov S., Hoffmann F., Kéry A., Renner E.D., Hartl D., Lohse P., Huss K., Fraunberger P., Malley J.D., Zellerer S. (2006). Cytokine profile in PFAPA syndrome suggests continuous inflammation and reduced anti-inflammatory response. Eur. Cytokine Netw..

[14] Sangiorgi E., Rigante D. (2022). The clinical chameleon of autoinflammatory diseases in children. Cells.

[15] Jiao Y., Wu L., Huntington N.D., Zhang X. (2020). Crosstalk between gut microbiota and innate immunity and its implication in autoimmune diseases. Front. Immunol..

[16] Kettunen S., Lantto U., Koivunen P., Tapiainen T., Uhari M., Renko M. (2018). Risk factors for periodic fever, aphthous stomatitis, pharyngitis, and adenitis (PFAPA) syndrome: A case-control study. Eur. J. Pediatr..

[17] Notarbartolo V., Giuffrè M., Montante C., Corsello G., Carta M. (2022). Composition of human breast milk microbiota and its role in children’s health. Pediatr. Gastroenterol. Hepatol. Nutr..

[18] Na J.Y., Cho Y., Lee J., Yang S., Kim Y.J. (2022). Immune-modulatory effect of human milk in reducing the risk of Kawasaki disease: A nationwide study in Korea. Front. Pediatr..

[19] Guo M.M., Tsai I.H., Kuo H.C. (2021). Effect of breastfeeding for 6 months on disease outcomes in patients with Kawasaki disease. PLoS ONE.

[20] Gurbuz F., Canbulat Sahiner N., Unal E., Gurbuz A.S., Baysal T. (2022). Breastfeeding does not protect against the development of carditis in children with acute rheumatic fever. Cardiol. Young.

[21] Spatz D.L. (2012). Breastfeeding is the cornerstone of childhood nutrition. J. Obstet. Gynecol. Neonatal Nurs..

[22] Bagci Bosi A.T., Eriksen K.G., Sobko T., Wijnhoven T.M., Breda J. (2016). Breastfeeding practices and policies in WHO European Region Member States. Public Health Nutr..

[23] Rigante D. (2023). Febrile children with breaches in the responses of innate immunity. Expert Rev. Clin. Immunol..

[24] Magnano San Lio R., Maugeri A., La Rosa M.C., Cianci A., Panella M., Giunta G., Agodi A., Barchitta M. (2021). The impact of socio-demographic factors on breastfeeding: Findings from the “Mamma & Bambino” cohort. Medicina.

